# Association Between MRI-Defined Acromioclavicular Joint Degeneration and Supraspinatus Fatty Infiltration in Shoulders Without MRI-Visible Supraspinatus Tear

**DOI:** 10.3390/jimaging12100521

**Published:** 2026-10-19

**Authors:** Alex Alexiev, Katarzyna Bokwa Dąbrowska, Vladislavs Gordins, Katarina Nilsson Helander, Pawel Szaro

**Affiliations:** 1 Department of Radiology, Institute of Clinical Sciences, Sahlgrenska Academy, University of Gothenburg, SE-413 45 Gothenburg, Sweden; alex.alexiev@gu.se (A.A.); katarzyna.bokwa.dabrowska@gu.se (K.B.D.); 2 Department of Musculoskeletal Radiology, Sahlgrenska University Hospital, Region Västra Götaland, Göteborgsvägen 31, SE-431 80 Mölndal, Sweden; 3 Department of Orthopaedics, Institute of Clinical Sciences, Sahlgrenska Academy, University of Gothenburg, Göteborgsvägen 31, SE-431 80 Mölndal, Sweden; vladislavs.gordins@vgregion.se (V.G.); katarina.nilsson.helander@vgregion.se (K.N.H.); 4 Department of Orthopaedics, Sahlgrenska University Hospital, Region Västra Götaland, Göteborgsvägen 31, SE-431 80 Mölndal, Sweden

**Keywords:** acromioclavicular joint, supraspinatus muscle, fatty infiltration, rotator cuff, Goutallier classification, shoulder, magnetic resonance imaging, joint degeneration

## Abstract

Supraspinatus fatty infiltration can occur without MRI-visible supraspinatus tear, but its relationship with acromioclavicular joint degeneration on shoulder MRI is unclear. We evaluated whether MRI-defined acromioclavicular joint degeneration was associated with supraspinatus fatty infiltration in shoulders without MRI-visible supraspinatus tear or definite tendinopathy. This retrospective single-center cross-sectional study included 250 shoulders from 250 patients selected from 673 consecutive shoulder MRI examinations performed between 2020 and 2024. Two readers independently assessed fatty infiltration using the Goutallier classification and four prespecified acromioclavicular joint degenerative features, with disagreements resolved by consensus. Acromioclavicular joint degeneration was operationally defined as at least two of four features. Fatty infiltration was present in 92 shoulders (36.8%) and acromioclavicular joint degeneration in 173 (69.2%); fatty infiltration occurred in 49.1% with versus 9.1% without degeneration (*p* < 0.001). After adjustment for age, sex, and side, acromioclavicular joint degeneration remained associated with fatty infiltration (adjusted odds ratio, 5.44; 95% confidence interval, 2.32–14.40; *p* < 0.001). Findings were consistent across sensitivity analyses addressing alternative definitions, age, and MRI field strength. In this referral-based cohort, MRI-defined acromioclavicular joint degeneration and supraspinatus fatty infiltration frequently coexisted despite the absence of MRI-visible supraspinatus tear or definite tendinopathy.

## 1. Introduction

Fatty infiltration of the supraspinatus muscle is most often discussed in the context of rotator-cuff tendon tears. Fatty infiltration refers to fat accumulation within skeletal muscle and is often discussed in the context of fatty degeneration or myosteatosis. However, it is distinct from muscle atrophy, which reflects reduced muscle size, and from sarcopenia and sarcopenic obesity, which are broader clinical conditions involving muscle quantity and function. In the present study, fatty infiltration specifically refers to visually detectable intramuscular fat assessed using the Goutallier classification on T1-weighted MRI [1,2]. Fatty infiltration may also be detected without MRI-visible supraspinatus tear, a phenomenon that is less well understood. Previous studies have reported supraspinatus or rotator-cuff fatty infiltration in shoulders without tendon rupture, and increasing age appears to be an important associated factor [3,4]. In patients with rotator-cuff disease, fatty infiltration has been associated with muscle function, tendon healing, retear risk, and postoperative outcome [5,6,7]. In contrast, less is known about why fatty infiltration occurs when no supraspinatus tear is visible on MRI. Clarifying associated imaging findings in these patients may improve radiological interpretation and help avoid treating fatty infiltration as an isolated or unexplained muscle finding.

Acromioclavicular joint osteoarthritis is common on shoulder imaging and is frequently reported on routine MRI even in younger populations. Typical imaging features include joint-space narrowing, superior or inferior osteophytes, capsular hypertrophy, joint effusion or capsular distension, subchondral cysts, and bone-marrow edema. Inferior osteophytes and capsular hypertrophy may narrow the supraspinatus outlet or indent the supraspinatus muscle-tendon unit [8,9,10]. The prevalence of acromioclavicular joint osteoarthritis varies substantially between populations and imaging methods, and osteoarthritis may also be present in individuals not seeking care [11]. Therefore, acromioclavicular joint osteoarthritis may represent either a symptomatic degenerative process or an incidental imaging finding and may coexist with other shoulder abnormalities [12]. Previous studies have mainly examined fatty infiltration in relation to rotator-cuff tears, shoulder pain, impingement, or postoperative outcomes [6,7,13,14]. Direct data on whether acromioclavicular joint degeneration is associated with supraspinatus fatty infiltration in shoulders without MRI-visible supraspinatus tear or definite tendinopathy are limited. Establishing whether these findings cluster on MRI is relevant because both are commonly encountered, routinely assessable, and potentially influenced by age. Therefore, the purpose of this study was to evaluate whether MRI-defined acromioclavicular joint degeneration is associated with supraspinatus fatty infiltration in shoulders without MRI-visible supraspinatus tear or definite tendinopathy, after adjustment for age, sex, and side.

## 2. Materials and Methods

### 2.1. Study Design and Setting

This retrospective observational cross-sectional study was conducted at the radiology department of a tertiary musculoskeletal center. Consecutive shoulder magnetic resonance imaging (MRI) examinations performed between 2020 and 2024 at our institution were identified from the institutional radiology database. The study was approved by the Swedish Ethical Review Authority (protocol codes 2020-05954 [25 November 2020] and 2024-05543-1 [1 October 2024]).

### 2.2. Study Size and Participants

An a priori sample-size calculation was based on the planned primary unadjusted comparison between acromioclavicular joint degeneration and supraspinatus fatty infiltration using a 2 × 2 chi-square framework. Preliminary data from 20 examinations suggested an effect size of Cohen’s w = 0.18. At a two-sided α of 0.05 and 80% power, 243 shoulders were required. The final cohort comprised 250 shoulders. The primary association was subsequently evaluated using multivariable logistic regression with adjustment for prespecified covariates. The 20 examinations used for the preliminary effect-size estimate were included in the final cohort.

All examinations were re-evaluated for supraspinatus tendon integrity before inclusion by two musculoskeletal radiologists with 7 and 3 years of dedicated musculoskeletal imaging experience, respectively (K.B.D. and A.A.). Disagreements regarding eligibility were resolved by a third senior musculoskeletal radiologist (P.S.). Examinations were excluded when the supraspinatus tendon demonstrated a partial- or full-thickness tear or definite MRI tendinopathy, operationally defined as visually evident tendon thickening accompanied by markedly increased intratendinous signal intensity. Mild isolated intratendinous signal heterogeneity without definite tendon thickening was not considered sufficient for exclusion. This operational exclusion criterion was not equivalent to a formal tendinopathy severity scale, such as the four-grade MRI classification described by Sein et al. [15]. Eligibility assessment focused on the supraspinatus tendon. Abnormalities of the infraspinatus and subscapularis tendons were not additional exclusion criteria. Additional exclusion criteria were previous shoulder surgery or postoperative changes and incomplete or non-diagnostic imaging. The study included examinations performed using the routine shoulder MRI protocol rather than dedicated imaging protocols for suspected primary muscle disorders.

### 2.3. MRI Protocol

MRI examinations were performed at 1.5 T (Ingenia Ambition X, Philips; *n* = 63) or 3.0 T (Ingenia, Philips; *n* = 187) using dedicated shoulder coils. The routine protocol included axial oblique PD-weighted fat-suppressed, coronal oblique T2-weighted fat-suppressed, sagittal oblique T1-weighted, and sagittal oblique T2-weighted fat-suppressed sequences. Representative acquisition parameters are summarized in Table 1.

### 2.4. Image Assessment

Two radiologists with 10 and 3 years of musculoskeletal imaging experience independently re-evaluated all eligible MRI examinations (P.S., A.A.). A six-week washout period was maintained before the independent image assessment to minimize recall bias. Readers were blinded to clinical information, original MRI reports, and each other’s assessments. After completion of the independent readings, disagreements were resolved by consensus between the two readers. Consensus ratings were used for the main analyses, whereas the independent readings were used to calculate inter-reader agreement.

Supraspinatus fatty infiltration was assessed on oblique sagittal T1-weighted images at the scapular Y-view level using the Goutallier classification [1]. For the primary analysis, any visually detectable supraspinatus fatty infiltration was defined as Goutallier grade ≥ 1, whereas grade 0 was considered absent. Dichotomization was chosen because the primary objective was to identify factors associated with the presence of any visually detectable fatty infiltration rather than to model increasing severity across the full ordinal scale. This threshold was chosen to investigate early visible fatty infiltration in this selected cohort. Because the distinction between grades 0 and 1 may be subtle and grade ≥ 2 represents more established fatty infiltration, Goutallier grade ≥ 2 was evaluated as a secondary outcome.

Acromioclavicular joint degeneration was assessed using four prespecified MRI features that could be consistently evaluated across all examinations: clavicular bone-marrow edema, clavicular osteophytes, acromial bone-marrow edema, and acromial osteophytes. Each feature was recorded as absent or present, yielding a summed score from 0 to 4 representing the number of assessed degenerative MRI features rather than a validated measure of osteoarthritis severity. For the primary analysis, MRI-defined acromioclavicular joint degeneration was operationally defined as the presence of at least two of the four features. The threshold was selected a priori to reduce classification based on an isolated imaging finding. Because this operational definition has not been externally validated, robustness was assessed using alternative definitions and the summed 0–4 score. Bone-marrow edema was defined as focal subchondral marrow signal abnormality adjacent to the AC joint, hyperintense on fluid-sensitive fat-suppressed sequences relative to normal marrow. Osteophytes were defined as definite osseous marginal projections arising from the distal clavicle or adjacent acromion. Findings were assessed using the axial and coronal fluid-sensitive sequences, with sagittal images used when necessary for confirmation.

### 2.5. Variables

The primary outcome was the presence of supraspinatus fatty infiltration, defined as Goutallier grade ≥ 1. The primary exposure was MRI-defined acromioclavicular joint degeneration, operationally defined as the presence of at least two of four prespecified MRI features. Covariates included age, sex, and side of the examined shoulder. These covariates were selected a priori based on biological plausibility and availability across the entire cohort.

### 2.6. Bias

To reduce measurement bias, MRI examinations were assessed independently by two readers who were blinded to clinical information, original MRI reports, and each other’s assessments. Consecutive examinations were screened to minimize selective inclusion within the available clinical MRI population, and one shoulder per patient was included to preserve independence of observations. If a patient had bilateral or repeated shoulder MRI examinations during the study period, only the chronologically first eligible examination was included. However, because the source population consisted of patients referred for clinical shoulder MRI at a tertiary center, referral and selection bias cannot be excluded.

### 2.7. Statistical Analysis

Statistical analyses were performed using R version 4.3.3 (R Foundation for Statistical Computing, Vienna, Austria). Continuous variables were summarized as median with interquartile range, and categorical variables as *n* (%). Between-group comparisons used the Wilcoxon rank-sum test, Pearson’s chi-squared test, or Fisher’s exact test, as appropriate. Univariable logistic regression was used to estimate crude odds ratios with 95% confidence intervals. A prespecified multivariable logistic regression model including acromioclavicular joint degeneration, age, sex, and side was fitted to estimate the adjusted association with supraspinatus fatty infiltration. Age was modeled continuously and reported per 10-year increase. The final dataset contained 92 primary outcome events for four prespecified predictors in the multivariable model, corresponding to approximately 23 outcome events per predictor and providing an adequate number of events relative to model complexity. Variance inflation factors were examined and did not suggest problematic multicollinearity. Sensitivity analyses compared the primary ≥2-feature definition with alternative definitions including ≥1 feature, any osteophyte, and any osteophyte combined with bone-marrow edema. The summed 0–4 acromioclavicular joint degeneration score was additionally analyzed as an ordinal predictor. Age-related sensitivity analyses included comparison of the age distribution according to acromioclavicular joint degeneration status, analyses stratified at age 50 years, and an acromioclavicular joint degeneration × age interaction term. The age-stratified models were adjusted for sex and side. An additional sensitivity analysis included MRI field strength (1.5 T versus 3.0 T) as a covariate to assess whether the primary association was sensitive to differences in acquisition field strength. To assess whether the primary association was sensitive to the functional form of age, the multivariable model was additionally fitted with age modeled non-linearly using a natural cubic spline with three degrees of freedom and compared with the linear-age model using a likelihood-ratio test. A secondary analysis used Goutallier grade ≥ 2 fatty infiltration as the outcome; because of separation, Firth penalized logistic regression was applied. Inter-reader agreement was assessed using Cohen’s kappa and Gwet’s AC1 with 95% confidence intervals. Two-sided *p*-values < 0.05 were considered statistically significant.

## 3. Results

### 3.1. Study Cohort

**Figure 1:**
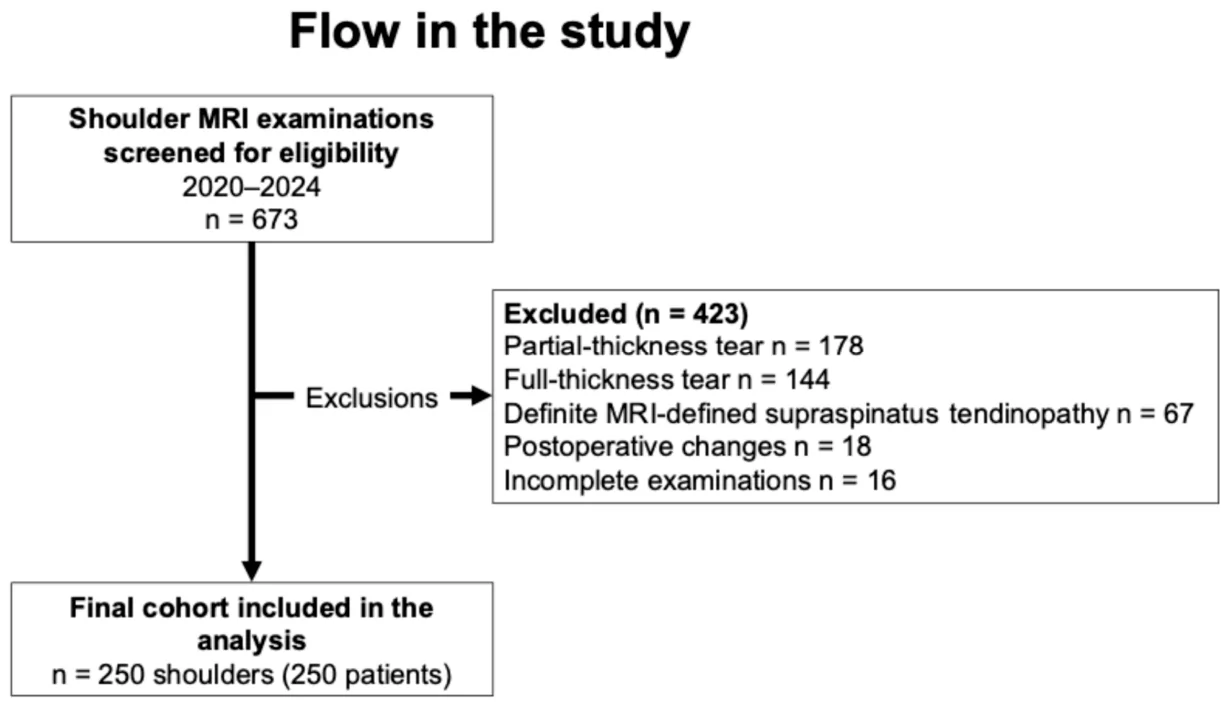
Patient selection flowchart.

A total of 673 shoulder MRI examinations were screened. After application of the exclusion criteria, 423 examinations were excluded because of partial-thickness supraspinatus tear (*n* = 178), full-thickness supraspinatus tear (*n* = 144), definite MRI-defined supraspinatus tendinopathy (*n* = 67), previous shoulder surgery or postoperative changes (*n* = 18), or incomplete or non-diagnostic imaging (*n* = 16) (Figure 1).

### 3.2. Inter-Reader Agreement

Inter-reader agreement for supraspinatus fatty infiltration was substantial (Cohen’s κ = 0.78; 95% CI, 0.71–0.86), with Gwet’s AC1 = 0.78 (95% CI, 0.71–0.86). For MRI-defined acromioclavicular joint degeneration, agreement was also substantial (Cohen’s κ = 0.74; 95% CI, 0.65–0.83), with Gwet’s AC1 = 0.83 (95% CI, 0.76–0.89). Agreement for individual acromioclavicular joint features ranged from κ = 0.65 to 0.82 and from Gwet’s AC1 = 0.67 to 0.83. Additional inter-reader agreement analyses are provided in Appendix A.

### 3.3. Supraspinatus Fatty Infiltration

**Figure 2:**
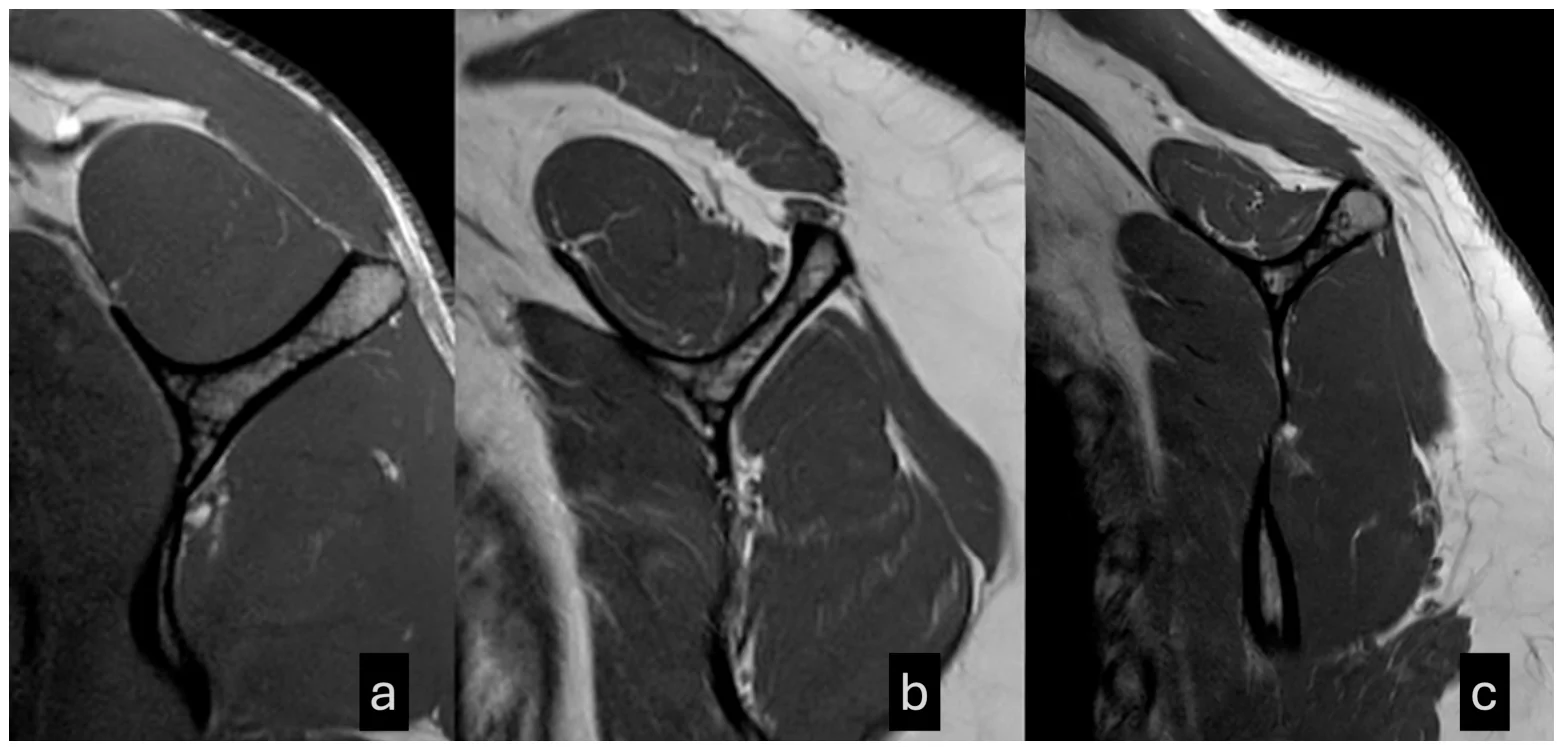
Representative oblique sagittal T1-weighted MR images of the supraspinatus muscle demonstrating Goutallier grade 0 (**a**), grade 1 (**b**), and grade 2 (**c**) fatty infiltration.

Supraspinatus fatty infiltration was present in 92/250 shoulders (36.8%) and absent in 158/250 shoulders (63.2%). Patients with fatty infiltration were older than those without fatty infiltration (median age 57 years, interquartile range 50–64, versus 42.5 years, interquartile range 31–51.8; *p* < 0.001) (Table 2). Sex distribution and side did not differ significantly between groups. The Goutallier grade distribution was grade 0 in 158/250 shoulders (63.2%), grade 1 in 67/250 shoulders (26.8%), and grade 2 in 25/250 shoulders (10.0%). No shoulders were classified as grade 3 or 4 (Figure 2).

### 3.4. Acromioclavicular Joint Degeneration

Acromioclavicular joint degeneration was present in 173/250 shoulders (69.2%) and was more frequent in shoulders with supraspinatus fatty infiltration than in those without fatty infiltration: 85/92 (92.4%) versus 88/158 (55.7%), respectively (*p* < 0.001) (Table 2). The most frequent individual features were clavicular and acromial osteophytes (Table 2). Among the 173 shoulders meeting the primary ≥2-feature definition, 118 (68.2%) demonstrated at least one osteophyte and at least one bone marrow edema feature, whereas 39 (22.5%) had osteophytes without bone-marrow edema and 16 (9.2%) had bone-marrow edema without osteophytes (Table A1).

Patients with acromioclavicular joint degeneration were older than those without acromioclavicular joint degeneration: median age 51 years (interquartile range, 44–59) versus 34 years (interquartile range, 24–48), respectively (*p* < 0.001).

### 3.5. Association Between Acromioclavicular Joint Degeneration and Supraspinatus Fatty Infiltration

**Figure 3:**
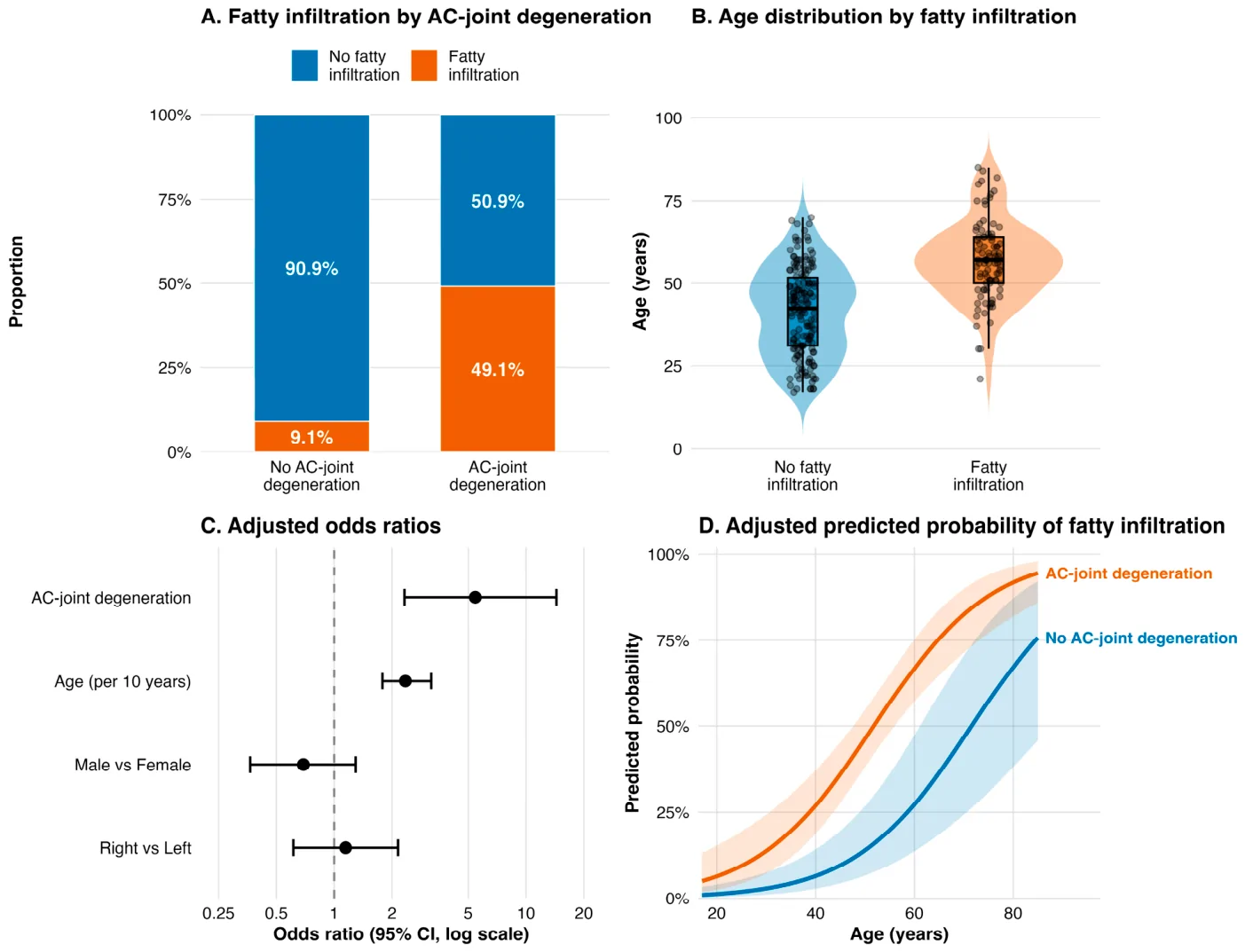
Association between acromioclavicular (AC) joint degeneration and supraspinatus fatty infiltration. (**A**) Proportion of shoulders with and without supraspinatus fatty infiltration according to AC-joint degeneration status. (**B**) Age distribution according to fatty infiltration status; points represent individual examinations, violins show the distribution, and boxplots indicate the median and interquartile range. (**C**) Adjusted odds ratios with 95% confidence intervals from the multivariable logistic regression model including AC-joint degeneration, age, sex, and side. (**D**) Adjusted marginal predicted probability of fatty infiltration with 95% confidence intervals across age, stratified by AC-joint degeneration status and standardized over the observed distributions of sex and side.

Cross-tabulation demonstrated a significant association between acromioclavicular joint degeneration and supraspinatus fatty infiltration (Table 3; Figure 3A). The association was significant by Pearson’s chi-squared test with Yates’ continuity correction (χ^2^ = 35.03, df = 1, *p* < 0.001).

In univariable logistic regression, MRI-defined acromioclavicular joint degeneration and older age were associated with higher odds of supraspinatus fatty infiltration (Table 4). In the multivariable model, acromioclavicular joint degeneration remained associated with fatty infiltration after adjustment for age, sex, and side (adjusted OR, 5.44; 95% CI, 2.32–14.40; *p* < 0.001). Older age was also independently associated with fatty infiltration (adjusted OR, 2.35 per 10-year increase; 95% CI, 1.78–3.21; *p* < 0.001), whereas sex and side were not significantly associated with the outcome (Table 4). These findings are illustrated by the higher frequency of fatty infiltration in shoulders with acromioclavicular joint degeneration (Figure 3A), the age distribution according to fatty infiltration status (Figure 3B), and the adjusted odds ratios (Figure 3C). The adjusted predicted probability of fatty infiltration increased with age in both groups but remained higher in shoulders with acromioclavicular joint degeneration across the examined age range (Figure 3D).

### 3.6. Sensitivity Analyses

Sensitivity analyses yielded findings consistent with the primary analysis. The association remained present when acromioclavicular joint degeneration was defined by the presence of any osteophyte (adjusted OR, 3.54; 95% CI, 1.69–7.86; *p* = 0.001) and when defined by any osteophyte combined with bone-marrow edema (adjusted OR, 3.51; 95% CI, 1.88–6.68; *p* < 0.001) (Table A2).

The summed 0–4 acromioclavicular joint degeneration score was also associated with fatty infiltration when analyzed as an ordinal predictor (adjusted OR, 2.00 per additional feature; 95% CI, 1.49–2.76; *p* < 0.001), although observed proportions were not strictly monotonic across individual score categories (Table A3).

Age-stratified analyses demonstrated associations both in patients younger than 50 years (adjusted OR, 6.67; 95% CI, 2.10–29.78; *p* = 0.004) and in those aged 50 years or older (adjusted OR, 7.73; 95% CI, 2.47–29.89; *p* = 0.001). There was no evidence of interaction between age and acromioclavicular joint degeneration (interaction OR, 1.29 per 10-year increase in age; 95% CI, 0.59–2.55; Wald *p* = 0.484). Allowing for a non-linear association between age and fatty infiltration did not improve model fit compared with the linear-age model (likelihood-ratio *p* = 0.990), and the estimated association between acromioclavicular joint degeneration and fatty infiltration was essentially unchanged (adjusted OR, 5.43; 95% CI, 2.30–14.53; *p* < 0.001) (Table A4).

Additional adjustment for MRI field strength did not materially alter the association between acromioclavicular joint degeneration and supraspinatus fatty infiltration (adjusted OR, 5.88; 95% CI, 2.45–16.07; *p* < 0.001). MRI field strength itself was not significantly associated with fatty infiltration (3.0 T versus 1.5 T: adjusted OR, 0.74; 95% CI, 0.34–1.59; *p* = 0.439), and inclusion of field strength did not significantly improve model fit (likelihood-ratio *p* = 0.438) (Table A4).

All 25 shoulders with Goutallier grade ≥ 2 fatty infiltration had MRI-defined acromioclavicular joint degeneration, resulting in complete separation. Firth penalized logistic regression was therefore used for this secondary analysis. The analysis supported the direction of the primary association, but the effect estimate was imprecise because of the limited number of grade ≥ 2 events and should not be interpreted as a precise estimate of effect magnitude (Table A5).

## 4. Discussion

Findings from this study demonstrate a consistent imaging association between acromioclavicular joint degenerative changes and supraspinatus fatty infiltration in a selected cohort without MRI-visible supraspinatus tear or definite tendinopathy. Supraspinatus fatty infiltration was substantially more frequent in shoulders with MRI-defined acromioclavicular joint degeneration than in those without it, despite the absence of MRI-visible supraspinatus tear or definite tendinopathy. After adjustment for age, sex, and side, acromioclavicular joint degeneration was associated with approximately fivefold higher odds of fatty infiltration. The association remained consistent across alternative definitions of acromioclavicular joint degeneration and sensitivity analyses addressing age, non-linear age effects, and MRI field strength. Age was also associated with fatty infiltration, but the relationship between acromioclavicular joint degeneration and fatty infiltration was not fully explained by age.

Previous CT arthrography and MRI studies have demonstrated that rotator-cuff fatty infiltration can occur despite preserved tendon integrity and becomes more frequent with increasing age [3,4]. Meghashyama et al. investigated factors associated with supraspinatus muscle atrophy in patients aged 50 years or older with atraumatic shoulder pain, including patients with no tear, partial-thickness tears, and full-thickness tears [6]. Acromioclavicular joint arthritis was evaluated as one of several potential predictors of supraspinatus atrophy, which was quantified using the occupation ratio, and no significant association was identified. In their subgroup of patients without rotator-cuff tears, the primary outcome remained supraspinatus atrophy. Thus, although their study included both acromioclavicular joint abnormalities and supraspinatus fatty infiltration, it did not specifically evaluate whether acromioclavicular joint degeneration was associated with supraspinatus fatty infiltration in shoulders with preserved supraspinatus tendon integrity. The present study addresses this distinct question, with fatty infiltration as the primary outcome and MRI-defined acromioclavicular joint degeneration as the primary exposure.

Advanced rotator-cuff fatty infiltration is generally considered poorly reversible. The significance of mild grade 1 changes in shoulders without MRI-visible supraspinatus tear remains uncertain [2,7,16]. Previous imaging studies suggest that acromioclavicular osteophytes may contribute to narrowing of the supraspinatus outlet [9]. However, because this study was cross-sectional, it cannot determine whether acromioclavicular joint degeneration precedes, follows, or simply coexists with supraspinatus fatty infiltration. Age was an important potential confounder because both acromioclavicular degeneration and supraspinatus fatty infiltration increased with age. Although patients with acromioclavicular joint degeneration were older, the association remained strong after age adjustment and in age-stratified analyses, was essentially unchanged when age was modeled non-linearly, and showed no evidence of interaction by age. The relatively high prevalence of MRI-defined acromioclavicular joint degeneration in our cohort is therefore not unexpected, given that AC-joint osteoarthritis is common even in asymptomatic shoulders and becomes more prevalent with age, although our broad operational definition and referral-based population may also have contributed [11]. Taken together, the age-adjusted, age-stratified, interaction, and non-linear sensitivity analyses suggest that the observed association was not fully accounted for by the modeled effects of age (Table A4). Nevertheless, residual age-related confounding cannot be excluded. Other age-related or structural shoulder factors, including glenohumeral osteoarthritis and glenoid or scapular morphology, may also contribute to rotator-cuff muscle degeneration and were not evaluated in the present study.

Several explanations for the observed association are possible. Acromioclavicular osteophytes may alter the anatomical relationship between the acromioclavicular joint and the supraspinatus outlet [8,9]. However, the present study did not assess outlet dimensions, acromiohumeral distance, subacromial–subdeltoid bursitis, direct mass effect on the supraspinatus muscle-tendon unit, or the direction of individual osteophytes. Consequently, the contribution of subacromial structural factors and a potential mechanical impingement mechanism cannot be determined. Alternatively, acromioclavicular joint degeneration and supraspinatus fatty infiltration may represent parallel manifestations of a broader age-related or degenerative shoulder phenotype [3,4,11,12]. The present findings should therefore be interpreted as evidence of an imaging association rather than proof that AC-joint degeneration contributes directly to supraspinatus fatty infiltration.

The immediate relevance of these findings is primarily radiological rather than therapeutic in the current state of knowledge. In shoulders without MRI-visible supraspinatus tear, supraspinatus fatty infiltration frequently coexisted with acromioclavicular joint degenerative features. Awareness of this association may help radiologists interpret supraspinatus fatty infiltration within the broader pattern of shoulder degeneration rather than automatically attributing it to primary supraspinatus tendon pathology [5,13,14,17]. However, the present data do not establish whether the acromioclavicular findings are symptomatic, whether they contribute mechanistically to muscle changes, or whether their treatment would influence symptoms or progression.

Supraspinatus fatty infiltration was assessed using the Goutallier classification and categorized as absent or present in the primary analysis. Grade ≥ 1 was chosen to capture early visible fatty infiltration in this selected cohort. The relatively high frequency of grade 1 findings indicates that mild fatty infiltration was common in this referral-based MRI cohort even in the absence of MRI-visible supraspinatus tendon tear. We acknowledge that the distinction between grades 0 and 1 may be subtle and potentially subjective. However, inter-reader agreement was substantial and analyses restricted to grade ≥ 2 fatty infiltration yielded similar directional findings. Because grade ≥ 2 represents more established fatty infiltration, it was evaluated as a secondary outcome. Although the number of AC-joint degenerative features was also analyzed as an ordinal predictor, neither analysis provides a comprehensive assessment of the relationship between osteoarthritis severity, fatty infiltration severity, and subacromial structural abnormalities. All MRI examinations were reviewed independently by two blinded readers, disagreements were resolved by consensus, and inter-reader agreement was assessed using both Cohen’s kappa and Gwet’s AC1 [18,19,20]. Future studies using quantitative MRI techniques, such as Dixon-based fat-fraction imaging, may provide a more sensitive and reproducible assessment of subtle fatty infiltration [17,21].

Several limitations should be acknowledged. The definition of acromioclavicular joint degeneration was an operational MRI-based composite developed for the present study rather than an externally validated diagnostic or severity classification. More comprehensive systems, such as the MAC score proposed by Farkhondeh Fal et al. [22], incorporate additional morphological measurements that were not evaluated in our cohort. Consequently, our findings should be interpreted as applying specifically to the assessed MRI pattern of degenerative features and may not be directly generalizable to clinical, radiographic, or surgical definitions of acromioclavicular joint degeneration. The retrospective single-center design may introduce selection bias, as the cohort included patients referred for clinical shoulder MRI. The cross-sectional design precludes conclusions about temporal sequence or causality. Clinical and imaging variables such as pain duration, shoulder function, activity level, occupation, body mass index, diabetes mellitus, HbA1c, glenohumeral osteoarthritis, and shoulder morphology were not evaluated. Neuropathy and myopathy were also not systematically assessed or excluded and may independently contribute to supraspinatus muscle changes. Consequently, residual confounding cannot be excluded. Fatty infiltration was assessed visually using the semi-quantitative Goutallier classification rather than quantitative fat-fraction MRI. Finally, subtle tendinopathic or microstructural supraspinatus tendon abnormalities may have remained despite exclusion of MRI-visible tears and definite tendinopathy. Concomitant abnormalities of the other rotator-cuff tendons were not systematically analyzed and may also have influenced supraspinatus muscle changes. The tendinopathy exclusion was based on qualitative MRI assessment rather than standardized tendinopathy grading or quantitative tendon-thickness measurements. Although the observed association was consistent across multiple sensitivity analyses, the study should be interpreted primarily as hypothesis-generating. Confirmation in independent cohorts using externally validated definitions of acromioclavicular joint degeneration and quantitative MRI measures of muscle fat content is warranted.

Prospective longitudinal studies incorporating quantitative fat-fraction MRI, detailed tendon assessment, and standardized clinical phenotyping are needed to determine whether acromioclavicular joint degeneration precedes, accompanies, or follows supraspinatus fatty infiltration. Such studies should clarify whether both findings progress together or represent parallel manifestations of shoulder degeneration [5,13,14,17,21].

## 5. Conclusions

In shoulders without MRI-visible supraspinatus tear, MRI-defined acromioclavicular joint degeneration was associated with substantially higher odds of supraspinatus fatty infiltration after adjustment for age, sex, and side. The association was consistent across alternative definitions of acromioclavicular joint degeneration and sensitivity analyses addressing age and MRI field strength. These findings indicate that acromioclavicular joint degeneration and supraspinatus fatty infiltration frequently coexist on MRI in shoulders without MRI-visible supraspinatus tear or definite tendinopathy. Longitudinal studies using quantitative muscle-fat assessment are needed to determine their temporal and potential mechanistic relationship.

## Figures and Tables

**Table 1:** Shoulder MRI acquisition parameters for the examinations included in the study.

Field Strength	Sequence and Plane	TR (ms)	TE (ms)	Slice Thickness/Gap (mm)	FOV (mm)	Acquisition Matrix	NEX	ETL
1.5 T	Axial oblique PD-weighted TSE SPAIR	4141	25	3.0/0.3	180 × 180	300 × 300	1.9	14
	Coronal oblique T2-weighted TSE SPAIR	3611	55	3.0/0.3	160–200 × 160–200	228–288 × 228–288	3.8	20
	Sagittal oblique T1-weighted TSE	515–559	10	3.0/0.3	160 × 160	268 × 266	1.0	4
	Sagittal oblique T2-weighted TSE SPAIR	3000	50	3.0/0.3	140 × 140	200 × 200	2.0	15
3.0 T	Axial oblique PD-weighted TSE SPAIR	2700	30	3.0/0.5	140 × 140	280 × 280	1.3	10
	Coronal oblique T2-weighted TSE SPAIR	3000	45	3.0/0.5	140 × 140	268 × 268	1.4	11
	Sagittal oblique T1-weighted TSE	500–571	10.7–11.2	3.0/0.3	160 × 160	532 × 349	2.0	3
	Sagittal oblique T2-weighted TSE SPAIR	3382	70	3.0/0.5	140 × 140	280 × 280	1.4	12

Abbreviations: PD, proton density; TSE, turbo spin echo; SPAIR, spectral attenuated inversion recovery; FOV, field of view; NEX, number of excitations/averages; ETL, echo train length.

**Table 2:** Baseline characteristics stratified by supraspinatus fatty infiltration in shoulders without MRI-visible supraspinatus tear or definite tendinopathy (*n* = 250).

Variable	No Fatty Infiltration, *n* = 158	Fatty Infiltration, *n* = 92	*p*-Value *	Statistical Test
Age, years	42.5 (31, 51.8)	57 (50, 64)	<0.001	Wilcoxon rank-sum test
Sex			0.609	Pearson’s chi-squared test
Female	72 (45.6%)	45 (48.9%)		
Male	86 (54.4%)	47 (51.1%)		
Side			0.468	Pearson’s chi-squared test
Left	71 (44.9%)	37 (40.2%)		
Right	87 (55.1%)	55 (59.8%)		
Acromioclavicular joint degeneration, ≥2 features	88 (55.7%)	85 (92.4%)	<0.001	Pearson’s chi-squared test
Clavicular bone-marrow edema	53 (33.5%)	49 (53.3%)	0.002	Pearson’s chi-squared test
Clavicular osteophytes	72 (45.6%)	68 (73.9%)	<0.001	Pearson’s chi-squared test
Acromial bone-marrow edema	44 (27.8%)	52 (56.5%)	<0.001	Pearson’s chi-squared test
Acromial osteophytes	66 (41.8%)	58 (63.0%)	0.001	Pearson’s chi-squared test
Number of AC-joint degeneration features	2 (0, 2)	2 (2, 3)	<0.001	Wilcoxon rank-sum test

Abbreviations: AC, acromioclavicular. * *p*-values are reported to three decimal places, with values below 0.001 reported as <0.001. Values are presented as median (interquartile range) or *n* (%). *p*-values are descriptive and were not adjusted for multiple comparisons.

**Table 3:** Cross-tabulation of acromioclavicular joint degeneration and supraspinatus fatty infiltration.

Acromioclavicular Joint Degeneration	No Fatty Infiltration (*n*)	Fatty Infiltration (*n*)	Total (*n*)	Fatty Infiltration, %
No, <2 features	70	7	77	9.1%
Yes, ≥2 features	88	85	173	49.1%
Total	158	92	250	36.8%

**Table 4:** Univariable and multivariable logistic regression analyses for supraspinatus fatty infiltration.

Variable	Univariable OR	Univariable 95% CI	Univariable *p*-Value	Adjusted OR *	Adjusted 95% CI	Adjusted *p*-Value
Acromioclavicular joint degeneration, ≥2 features	9.66	4.47–24.16	<0.001	5.44	2.32–14.40	<0.001
Age, per 10 years	2.58	1.99–3.45	<0.001	2.35	1.78–3.21	<0.001
Male sex	0.87	0.52–1.46	0.609	0.69	0.37–1.29	0.251
Right side	1.21	0.72–2.05	0.468	1.15	0.61–2.15	0.667

* Multivariable model adjusted for acromioclavicular joint degeneration, age, sex, and side. Abbreviations: CI, confidence interval; OR, odds ratio.

## Data Availability

The datasets generated and/or analyzed during the current study are not publicly available because of patient privacy and the conditions of ethical approval, but they are available from the corresponding author upon reasonable request and subject to applicable ethical and data-protection requirements.

## Abbreviations

The following abbreviations are used in this manuscript:

AC	Acromioclavicular
AC1	Gwet’s agreement coefficient 1
BME	bone marrow edema
CI	confidence interval
IQR	interquartile range
MRI	magnetic resonance imaging
OR	odds ratio
PD	proton density
SPAIR	spectral attenuated inversion recovery
T1	T1-weighted imaging
T2	T2-weighted imaging
TE	echo time
TR	repetition time
TSE	turbo spin echo

## Appendix A. Additional Agreement, Feature Composition, and Sensitivity Analyses

For advanced fatty infiltration, defined as Goutallier grade ≥ 2, Cohen’s κ was 0.57 (95% CI, 0.39–0.72), and Gwet’s AC1 was 0.90 (95% CI, 0.85–0.94). For exact three-category grading (0, 1, and ≥2), Cohen’s κ was 0.68 (95% CI, 0.60–0.76), and Gwet’s AC1 was 0.74 (95% CI, 0.67–0.81). The difference between Cohen’s κ and Gwet’s AC1 for the grade ≥ 2 outcome likely reflects the low prevalence of advanced fatty infiltration and the resulting sensitivity of κ to imbalanced marginal distributions.

To further characterize the study-specific ≥2-feature definition of acromioclavicular joint degeneration, the combinations of MRI features contributing to the primary exposure were examined. Among the 173 shoulders meeting the primary definition, 118 (68.2%) demonstrated at least one osteophyte and at least one bone marrow edema feature, 39 (22.5%) had osteophytes without bone-marrow edema, and 16 (9.2%) had bone-marrow edema without osteophytes (Table A1).

**Table A1:** Composition of MRI features among shoulders meeting the primary ≥2-feature definition of acromioclavicular joint degeneration (*n* = 173).

Number of Features	Feature Combination	*n* (%)
2	Clavicular osteophyte + acromial osteophyte	39 (22.5%)
2	Clavicular osteophyte + acromial BME	18 (10.4%)
2	Clavicular BME + acromial BME	16 (9.2%)
2	Clavicular BME + clavicular osteophyte	15 (8.7%)
2	Acromial BME + acromial osteophyte	12 (6.9%)
2	Clavicular BME + acromial osteophyte	11 (6.4%)
3	Clavicular BME + clavicular osteophyte + acromial osteophyte	14 (8.1%)
3	Clavicular osteophyte + acromial BME + acromial osteophyte	10 (5.8%)
3	Clavicular BME + clavicular osteophyte + acromial BME	8 (4.6%)
3	Clavicular BME + acromial BME + acromial osteophyte	4 (2.3%)
4	All four features (clavicular BME, clavicular osteophyte, acromial BME, and acromial osteophyte)	26 (15.0%)

Abbreviation: BME, bone-marrow edema. Percentages are calculated among the 173 shoulders meeting the primary ≥2-feature definition.

Additional sensitivity analyses were performed to assess the robustness of the association between acromioclavicular joint degeneration and supraspinatus fatty infiltration. The association remained present across alternative definitions of acromioclavicular joint degeneration, including at least one feature, at least two features, any osteophyte, and any osteophyte combined with bone-marrow edema (Table A2).

**Table A2:** Primary and alternative definitions of acromioclavicular joint degeneration.

Definition	Adjusted OR	95% CI	*p*-Value
Alternative definition: ≥1 feature	3.90	1.55–11.34	0.006
Primary definition: ≥2 features	5.44	2.32–14.40	<0.001
Any osteophyte	3.54	1.69–7.86	0.001
Any osteophyte plus any bone-marrow edema	3.51	1.88–6.68	<0.001

Abbreviations: CI, confidence interval; OR, odds ratio.

The summed acromioclavicular joint degeneration score was also analyzed as an ordinal predictor. When modeled as an ordinal predictor, each additional AC-joint feature was associated with higher odds of fatty infiltration (adjusted OR, 2.00 per feature; 95% CI, 1.49–2.76) (Table A3). Observed proportions were higher overall among scores ≥ 2, although the prevalence did not increase monotonically across every individual score category. The non-monotonic pattern between scores 0 and 1 likely reflects small subgroup sizes and should not be interpreted as evidence of a threshold effect.

**Table A3:** Acromioclavicular joint degeneration score analysis.

Analysis	Acromioclavicular Joint Degeneration Score	*n*	Fatty Infiltration, *n*	%	Adjusted OR	95% CI	*p*-Value
Ordinal model	Per additional feature				2.00	1.49–2.76	<0.001
Observed frequency	Score 0	49	6	12.2%			
Observed frequency	Score 1	28	1	3.6%			
Observed frequency	Score 2	111	45	40.5%			
Observed frequency	Score 3	36	24	66.7%			
Observed frequency	Score 4	26	16	61.5%			

Abbreviations: CI, confidence interval; OR, odds ratio.

Patients with acromioclavicular joint degeneration were older than those without acromioclavicular joint degeneration. However, age-stratified analyses showed consistent associations, and there was no evidence that age modified the association between acromioclavicular joint degeneration and supraspinatus fatty infiltration (Table A4).

**Table A4:** Sensitivity analyses addressing age and MRI field strength.

Analysis	Group or Effect	*n*	Age, Median (IQR)	Adjusted OR	95% CI	*p*-Value
Age distribution	AC-joint degeneration <2 features	77	34 (24, 48)	—	—	—
Age distribution	AC-joint degeneration ≥2 features	173	51 (44, 59)	—	—	<0.001 *
Age-stratified	AC-joint degeneration effect, age <50 years	133	—	6.67	2.10–29.78	0.004
Age-stratified	AC-joint degeneration effect, age ≥50 years	117	—	7.73	2.47–29.89	0.001
Interaction	AC-joint degeneration × age, per 10-year increase	250	—	1.29	0.59–2.55	0.484 †
Interaction	Likelihood-ratio test for interaction	250	—	—	—	0.495
Non-linear age model	AC-joint degeneration effect, age modeled with natural cubic spline	250	—	5.43	2.30–14.53	<0.001
MRI field-strength sensitivity analysis	AC-joint degeneration effect additionally adjusted for MRI field strength	250	—	5.88	2.45–16.07	<0.001
MRI field strength	3.0 T versus 1.5 T	250	—	0.74	0.34–1.59	0.439

Abbreviations: AC, acromioclavicular; CI, confidence interval; IQR, interquartile range; OR, odds ratio. * *p*-value from the Wilcoxon rank-sum test comparing age distributions between AC-joint degeneration groups. † Wald *p*-value for the interaction coefficient.

Age-stratified models were adjusted for sex and side. The interaction model included AC-joint degeneration, age (per 10-year increase), sex, side, and the AC-joint degeneration × age interaction term. The non-linear age model included AC-joint degeneration, age modeled using a natural cubic spline with three degrees of freedom, sex, and side. Comparison with the corresponding linear-age model showed no improvement in model fit (likelihood-ratio *p* = 0.990). The MRI field-strength sensitivity model additionally included field strength (1.5 T versus 3.0 T) as a covariate. Inclusion of MRI field strength did not significantly improve model fit (likelihood-ratio *p* = 0.438).

In the secondary analysis using Goutallier grade ≥ 2 as the outcome, all grade ≥ 2 cases occurred in shoulders with acromioclavicular joint degeneration. Because this resulted in separation, Firth penalized logistic regression was used. This analysis supported the direction of the primary findings but was imprecise because of the limited number of grade ≥ 2 cases and the wide confidence interval (Table A5).

**Table A5:** Sensitivity analysis using Goutallier grade ≥ 2 fatty infiltration.

Variable	Firth Odds Ratio	95% Confidence Interval	*p*-Value
Acromioclavicular joint degeneration ≥2 features	22.83	2.96–2938.34	<0.001
Age, per 10 years	1.22	0.87–1.72	0.243
Male sex	0.44	0.18–1.04	0.062
Right side	0.42	0.17–0.99	0.047

The multivariable Firth penalized logistic regression model included AC-joint degeneration, age, sex, and side.

Estimates for secondary covariates in the Firth model should be considered exploratory because of the limited number of grade ≥ 2 events.
